# Microengineered filters for efficient delivery of nanomaterials into mammalian cells

**DOI:** 10.1038/s41598-022-08300-2

**Published:** 2022-03-14

**Authors:** Dorsa Morshedi Rad, Meysam Rezaei, Payar Radfar, Majid Ebrahimi Warkiani

**Affiliations:** 1 School of Biomedical Engineering, University of Technology Sydney, Sydney, NSW 2007 Australia; 2 Genea, Sydney, NSW 2000 Australia; 3 Institute for Biomedical Materials and Devices (IBMD), Faculty of Science, University of Technology Sydney, Sydney, NSW 2007 Australia; 4 SUStech-UTS Joint Research Centre for Biomedical Materials and Devices, Southern University of Science and Technology, Shenzhen, 518055 People’s Republic of China; 5 Institute of Molecular Medicine, Sechenov University, Moscow, Russia 119991

**Keywords:** Biological techniques, Biotechnology

## Abstract

Intracellular delivery of nanomaterials into the cells of interest has enabled cell manipulation for numerous applications ranging from cell-based therapies to biomedical research. To date, different carriers or membrane poration-based techniques have been developed to load nanomaterials to the cell interior. These biotools have shown promise to surpass the membrane barrier and provide access to the intracellular space followed by passive diffusion of exogenous cargoes. However, most of them suffer from inconsistent delivery, cytotoxicity, and expensive protocols, somewhat limiting their utility in a variety of delivery applications. Here, by leveraging the benefits of microengineered porous membranes with a suitable porosity, we demonstrated an efficient intracellular loading of diverse nanomaterials to different cell types based on inducing mechanical disruption to the cell membrane. In this work, for the first time, we used ultra-thin silicon nitride (SiN) filter membranes with uniform micropores smaller than the cell diameter to load impermeable nanomaterials into adherent and non-adherent cell types. The delivery performance using SiN microsieves has been validated through the loading of functional nanomaterials from a few nanometers to hundreds of nanometers into mammalian cells with minimal undesired impacts. Besides the high delivery efficiency and improved cell viability, this simple and low-cost approach offers less clogging and higher throughput (10^7^ cell min^−1^). Therefore, it yields to the efficient introduction of exogenous nanomaterials into the large population of cells, illustrating the potential of these microengineered filters to be widely used in the microfiltroporation (MFP) setup.

## Introduction

Introducing synthetic biomolecules and nanoparticles into the cell cytosol, coined as intracellular delivery, is a necessary step in a growing number of applications ranging from cell-based therapy to genome-editing and probing intracellular space^1,2^. Current intracellular delivery methods are categorized into either carrier or membrane-poration based techniques^3^. In the former approach, carriers (e.g., viral vectors, cell ghosts, and liposomes) pass through the cell membrane via endocytosis or fusion pathway to achieve substantial cargo loading into the target cells^4,5^. However, delayed unpackaging, endosomal entrapment of cargo, recycling the cargo back to the cell exterior, and cytotoxicity involved in carrier-mediated delivery approaches are critical concerns for a majority of delivery applications^6,7^. These limitations have broadly motivated the development of membrane poration-based strategies to break through the target cell membranes via direct penetration or transient permeabilization^5,8^. By applying mechanical tension (mechanoporation)^9^, electric potential (electroporation)^10^, thermal deviation (thermoporation)^11^, laser-induced shock waves (optoporation)^12^, or biochemical pore-forming agents^13^, a multitude of nanopores could be generated in the plasma membrane. These approaches make the cells transiently permeable, allowing the passage of the exogenous nanomaterials to the intracellular space^14^. Electroporation is the most widely-used category of membrane poration method owing to its high delivery efficiency even for hard-to-transfect primary cells^15,16^. However, major challenges with the electroporation are cell death post-treatment and poor loading efficiency of large-sized biomolecules such as CRISPR/Cas RNPs and plasmid DNA^17,18^. Since this strategy tends to create membrane ruptures with a diameter of less than 50 nm, electric pulses with the width of tens of milliseconds are required to generate larger pores in the cell membrane. High-voltage operating conditions will trigger temperature increase and induce excessive stress and damage to the target cells as well as delivery biomaterials, thus limiting their implementation in most of the electroporation protocols^19–21^.

Among membrane poration approaches, mechanical means of cell permeabilization have made significant progress in the universal delivery of various biomaterials while inducing tolerable damage to the target cells^10^. Mechanical permeabilization of phospholipid bilayers can be accomplished via fluid shear forces, osmotic pressure deviations, and passage of cells through the microconstrictions^3^. Although fluid shear-based permeabilization approaches such as syringe loading proved to be less invasive, controlling the range of shear stress in the aqueous phase is challenging to verify experimentally^22^. Constriction-based mechanisms such as microfluidic cell squeezing and MFP have shown the potential for efficient delivery of cargoes with minimal impact on cell function and viability^9,23^. In the microfluidic cell squeezing platform, cells experience rapid membrane disruptions as they pass through the microfabricated constrictions smaller than the target cell diameter^16^. While this approach offers simplicity and control over the membrane disruption process resulted in minimized cell death, it suffers from clogging issues and cell size dependency owing to specific device geometry lowering its practicality.

MFP is another example of constriction-based strategies that creates an entry point for exogenous cargoes by forcing the cells through the membrane filters with pores smaller than the cell diameter. MFP was first developed by Williams et al*.*, in which they used 12 μm thick track-etched polycarbonate (PC) filters with 5–18 μm micropores to load FITC-dextrans (3–500 kDa) into the Chinese hamster ovary (CHO) cells^24^. Despite the success of MFP in cargo delivery with a stated transfection efficiency above 50%, it received much less attention until 2018 when Yen et al*.* developed transmembrane internalization assisted by membrane filtration (TRIAMF). In this method, track-etched membrane filters were deployed to deliver the CRISPR/Cas9 ribonucleoprotein complex targeting β2-microglobulin gene into the human hematopoietic stem cells^23^. Although this method is yet to be optimized, it is anticipated that the MFP system may represent a potential easy-to-use, low-cost, and high-throughput approach for efficient nanomaterial loading into the cells of interest. While the microfluidic cell squeezing platform can process a limited number of cells due to the clogging issues, MFP is able to pass the cells through the micropores instantly with no indications of clogging at high throughput^25^.

In this paper, for the first time, we used SiN microsieves in the MFP setup for highly efficient cytosolic loading of nanomaterials into the mammalian cell lines without compromising the cell viability. Utilizing these microengineered filters, we successfully optimized the delivery conditions for fluorescently labeled dextrans up to 2000 kDa. Since microfabricated SiN microsieves are ultra-thin (~ 1 µm), highly biocompatible, and rigid, less pressure and mechanical deformation are induced to the cells while passing through the uniform micropores^26^. We investigated the delivery performance of these microsieves in loading various cargoes into different cell lines and compared the delivery efficiency and viability of treated cells with those of conventional MFP and electroporation. Based on this simple and cost-effective approach, we have achieved highly efficient delivery of different nanomaterials into the adherent and non-adherent cells with minimum cell loss. Taken together, MFP using SiN membranes with regularly spaced pores demonstrates promising characteristics (up to 94.4% delivery efficiency, 98% cell viability, and high throughput (10^7^ cell min^−1^)), which make it an attractive intracellular delivery platform that influences cell-based research.

## Materials and methods

### Microfabrication process

**Figure 1:**
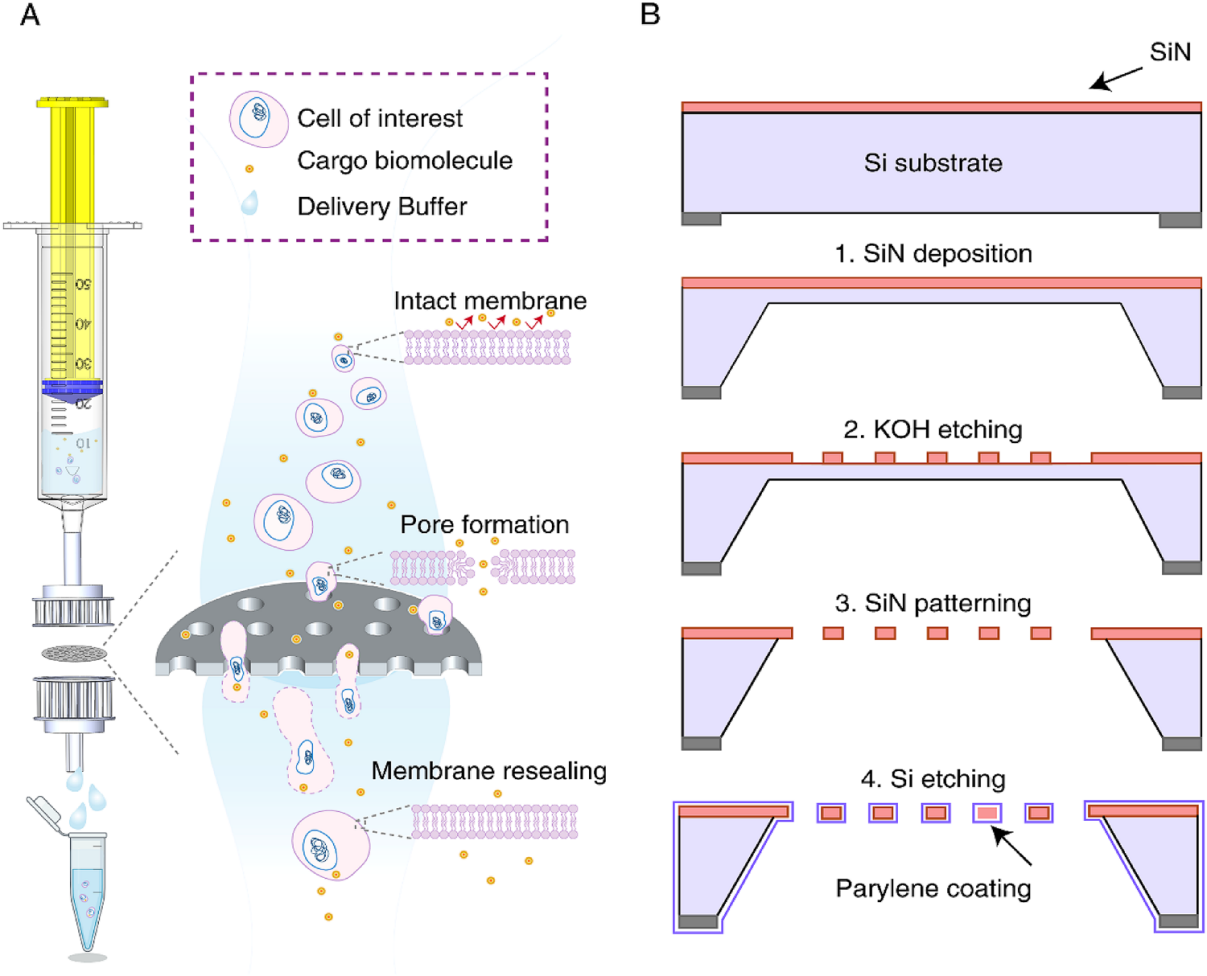
Schematic representation of the MFP delivery system. (**A**) In this platform, as the cells are forced through the microporous filters, they experience mechanical membrane deformations resulting in the generation of multiple discontinuities in the plasma membrane. These transient pores allow passive diffusion of exogenous nanomaterials across the plasma membrane. (**B**) Fabrication process of the SiN microsieves. First, SiN is coated on both sides of silicon (Si) wafers, followed by etching away the bulk Si using the KOH solution. Upon achieving the desired thickness, the surface of the wafers is patterned and then conformally coated with parylene to enhance the strength of the microsieves and reduce the cells attachment and prevent clogging issues. Figure (**A**) was generated using Biorender software (https://biorender.com/).

The SiN (Si_3_N_4_) membranes were fabricated using standard 100 mm, 480 μm thick double-sided polished silicon wafers (Fredricksburg, VA, USA). The fabrication process of the SiN microsieves has been performed in collaboration with Aquamarijn company (ZT, The Netherlands); details are shown in Fig. 1A,B. First, the Si substrate was deposited and pre-coated on both sides of wafers through low-pressure chemical vapor deposition at 850 °C. In the next step, the silicon wafers were dipped in potassium hydroxide (KOH) solution at 80 °C to etch away bulk silicon until the desired thickness for the SiN membrane was achieved. The surface of these wafers was patterned with Shipley’s positive photoresist (AZ P4330, Clariant Corp, USA) through the standard photolithography process. Next, an array of microholes with a circular pattern and size of 5 and 8 μm was etched onto the top SiN layer using reactive ion etching. This step is followed by dicing and etching the SiN wafers again in the KOH solution to remove the residuals and free the membrane. By etching away the residuals, once the membrane is freed, the SiN micropores were observed on the Si substrate. The exposed bottom side of the SiN layer was then removed by 4 h etching in the buffered oxide etch solution. Next, to enhance the strength of SiN microsieves as well as minimize the cell adhesion^27^, a thin layer of parylene was conformally deposited over these wafers.

### MFP system setup

Isopore track-etched PC membranes with 5 and 8 μm pore size and 13 mm polypropylene Swinnex filter holders were purchased from the Merck Millipore (MA, USA) and Sterlitech (WA, USA), respectively. Before the dead-end MFP, either PC or SiN membranes were mounted between two pieces of a 13 mm Swinnex filter holder, in which the top piece was connected directly to a syringe. To test the stability of the delivery setup and prime the microporous filters, 70% ethanol and sterilized DI water followed by PBS was flushed through a 3 ml syringe mounted on the syringe pump at the flow rate of 1 ml min^−1^. Next, the cell suspension was pumped through the microporous filter membranes by a syringe pump (KDS Legato, Sigma Aldrich Inc, MO, USA), and treated cells were collected in an Eppendorf tube for further assays.

### Fluid flow simulation

Additionally, to better understand the flow characteristics and fluid behavior passing through the micropores of SiN membranes, a computational fluid dynamic simulation was carried out using ANSYS Fluent 2019R3 (ANSYS Inc, Pennsylvania, USA). For simplification and lowering the computational load, only a portion of the actual MFP setup was modeled. The simulation was performed using a Laminar flow setting with a standard mesh and inlet velocity of 0.24 m s^−1^ at the inlet corresponding to the operating flow rate of the system. Furthermore, domain size and mesh independence checks were conducted to ensure the stability of the simulation results.

### Cell culture and viability assay

HeLa (cervical cancer cells, adherent) and THP1 (human acute monocytic leukemia cells, non-adherent) cell lines were purchased from ATCC (VA, USA) and cultured in DMEM and RPMI growth media, respectively, at 37 °C and 5% CO_2_. The growth media was supplemented with 10% FBS (Life Technologies Inc, CA, USA) and 1% penicillin–streptomycin (Thermo Fisher Scientific Inc, MA, USA). Prior to the delivery procedure, the viability of cells was characterized using the trypan blue exclusion assay (Lonza, MD, USA). In this assay, the cells were resuspended in PBS, and one part of the cell suspension was added to one part of 0.4% trypan blue dye. Next, a drop of the final solution was added to a hemocytometer, and the total number of unstained (viable) and stained (nonviable) cells, as well as the total number of cells, were counted. Then the viability of cells was calculated as:

$${\text{Cell}}\;{\text{viablity}}\% = \frac{{{\text{Number}}\;{\text{of}}\;{\text{unstained}}\;{\text{celles}} }}{{{\text{Total}}\;{\text{number}}\;{\text{of}}\;{\text{unstained}}\;{\text{and}}\;{\text{stained}}\;{\text{cells}}}} \times 100$$

### Delivery procedure using filter membranes

In the MFP platform, after centrifugation of cell suspension for 3 min at 500 g, the supernatant was discharged. As a proof of concept, a final concentration ranging from 5 × 10^6^- 1 × 10^7^ cells ml^−1^ was prepared and added to the delivery buffer and passed through the 40 μm cell strainers (Corning Inc, NY, USA) to remove cell clumps from the suspension. Accordingly, DPBS, DMEM, and optiMEM were used as a delivery buffer to optimize the experimental conditions. Before loading the cell suspension into the syringes, the delivery biomolecules were added to the solution. Next, the cell suspension was forced through the microengineered porous membranes using a syringe pump (Chemyx Inc, TX, USA) at a flow rate range between 0.5 to 10 ml min^−1^. First, we used Fluorescein isothiocyanate (FITC) dextran molecules (70 and 2000 kDa) (Sigma Aldrich, MO, USA) as delivery cargoes. Next, to prove the ability of this platform in loading large-sized cargoes, histone H2B-GFP (green fluorescent protein) plasmid DNA (Addgene plasmid # 11,680; RRID:Addgene_11680, MA, USA), kindly gifted by Geoff Wahl, was used as a delivery biomolecule to be loaded into the cells of interest^28^. Triplicate sampling was conducted for each data point.

### Cell membrane injury analysis

Next, the effect of MFP treatment on plasma membrane damage was carried out in the culture supernatant 24 h post-delivery. Accordingly, lactate dehydrogenase (LDH) assay was performed for both control and microfiltroporated samples using the pierce LDH cytotoxicity assay kit (Thermo Fisher Scientific Inc, MA, USA). The LDH activity was then calculated based on the absorption of culture supernatant at 490 and 680 nm according to the manufacturer's recommendation using the Varioskan Lux Reader (Thermo Fisher Scientific Inc, MA, USA).

### Quantitative polymerase chain reaction (qPCR) of cellular damage indicators

Immediately after the MFP treatment, total RNA was isolated from the treated and untreated cells using the PureLink ™ RNA Mini kit (Thermo Fisher Scientific Inc, MA, USA). The concentration and OD 260/280 ratio of the RNA samples were then measured using the NanoDrop ™ one/one microvolume UV–Vis spectrophotometer (Thermo Fisher Scientific Inc, MA, USA). Next, 1.0 µg of the RNA samples was used to evaluate the expression level of the DNA damage-inducible transcript 3 (DDIT3) and mitogen-activated protein kinase 14 (MAPK14) genes as indicators of cell injury and environmental stress using SuperScript ™ III platinum SYBR green one-step qPCR kit (Thermo Fisher Scientific Inc, MA, USA).

### Flow cytometry and cell recovery assessment

After MFP, the cells were collected in an Eppendorf tube, centrifuged at 500 g for 3 min, and resuspended in flow cytometry buffer, which contains PBS (Thermo Fisher Scientific Inc, MA, USA) supplied with 3% FBS and 1% Pluronic F-68 (Sigma Aldrich Inc, MO, USA). Quantitative analysis of biomolecule delivery to the cell interior coined as delivery efficiency was performed using the BD FACS LSR Fortessa flow cytometer, and data analysis was carried out using BD FACSDiva™ software (Becton–Dickinson, MA, USA). To assess the delivery efficiency quantitatively, we measured the mean fluorescent intensity of individual cells using flow cytometry. To this aim, we defined a threshold of 5% as a baseline to exclude endocytosis control cells, autofluorescence, and undesired binding of 70 kDa FITC-dextran to the cell surface^9^. Then, we defined the delivery efficiency as a fraction of live cells with fluorescent signals above this threshold. The viability of cells post-treatment was characterized by Sytox blue dead cell staining (Thermofisher Ins, MA, USA) and FITC Annexin V (Biolegned, CA, USA) to exclude dead and apoptotic cells from the analysis, respectively, according to the manufacturer’s protocol. Recovery of cells post MFP process was evaluated by counting the number of viable cells in the treated and untreated sample with the same number of cells seeded in the same volume via this equation:

$${\text{Cell}}\;{\text{recovery}}\% = \frac{{{\text{Number}}\;{\text{of}}\;{\text{viable}}\;{\text{celles}}\;{\text{in}}\;{\text{microfiltroporated}}\;{\text{sample}}}}{{{\text{Average}}\;{\text{number}}\;{\text{of}}\;{\text{viable}}\;{\text{cells}}\;{\text{from}}\;{\text{untreated}}\;{\text{sample}}}}$$

### Microscopic assessment

The microfiltroporated cells were seeded into a 6-well plate containing the complete media for the fluorescent and confocal microscopy analysis using the Nikon A1R inverted microscope 24 h post-treatment. FITC-labeled phalloidin (Sigma Aldrich Inc, MO, USA) and Hoechst 33,342 (Thermofisher Ins, MA, USA) stains were used according to the manufacturer’s protocol to visualize cytoskeletal actin and nucleus, respectively. For scanning electron microscopy (SEM), the treated cells were further processed to visualize the pores on the cell membrane compared to the untreated ones using the Zeiss Supr 55VP scanning electron microscope.

### Comparison of MFP delivery with the mainstream options

To evaluate the delivery performance of MFP using SiN microsieves, the loading efficiency and cell viability were compared with those of MFP via PC membranes, and conventional electroporation and lipofection (as representatives of benchtop methods). MFP using the PC membranes was performed in the same conditions (i.e., cell density and delivery buffer), and electroporation and lipofection were carried out using the Neon Transfection System (Thermo Fisher Scientific, MA, USA) and Lipofectamine™ 3000 Transfection Reagent (Thermo Fisher Scientific, MA, USA), respectively, according to the protocols recommended by the manufacturer.

### Statistical analysis

The statistical analysis was conducted using the unpaired student T-test and one-way analysis of variance (ANOVA) method, followed by Tukey’s multiple comparison test to evaluate whether a significant difference exists between the tested groups using GraphPad Prism software 6. *P*-values less than or equal to 0.05 were considered statistically significant, which are displayed as *P-value < 0.05, **P-value < 0.01, ***P-value < 0.001, and ****P-value < 0.0001.

## Results and discussion

### Optimizations of delivery conditions using track-etched PC membrane

MFP using track-etched PC membranes has been previously employed to load various cargoes (i.e., FITC-dextran and CRISPR-Cas9 RNP complex) inside different cell types. However, the optimal delivery conditions have not been heavily studied yet^23,24^. To close this gap, we decided to optimize the parameters affecting nanomaterial delivery using this platform. To establish the optimal delivery conditions, we tested the parameters that might influence both delivery efficiency and cell viability, including delivery buffer and operational flow rate used to push the cells through the filter membranes. Prior studies have shown that 40–60% appears to be an optimal deformation range^25,29^.

Moreover, it has been reported that the cells traversing through these pores received a significant amount of plasma membrane permeabilization while maintaining cell viability^23^. Hence, the microporous filters with 8 µm pore size were deployed in optimization experiments as it is approximately equal to 50% deformation of the average diameter in HeLa cells (16 ± 1 um). In each experiment, we optimized just one of the variables while maintaining the other variables constant. As a starting point, we focused on commercial PC track-etched filters that were originally used in the first report^24^. We first tried to load 70 kDa FITC-dextran into HeLa cells as it represents a typical mid-sized protein (~ 12 nm) and is a commonly used marker of intracellular delivery. We hypothesized that the operational flow rate could impact nanomaterial delivery and cell health outcomes. Therefore, we used various flow rates ranging from 0.5 to 10 ml min^−1^ to force the cells through the micropores of track-etched PC membranes. We found that at the flow rate of 2 ml min^−1^ the plasma membrane of 61.9% of target cells was permeabilized while they were 84.0% viable (Supplementary Fig. 1A–E). This optimal flow rate resulted in a trade-off between delivery efficiency and cell recovery for the processing of HeLa cells. As can be found in Supplementary Fig. 1F, at flow rates higher than 2 ml min^−1^, the cell viability started to decrease so that at the flow rate of 10 ml min^−1^ only about 50% of the treated cells were viable. A decline in cell viability can be attributed to the higher level of hydrodynamic shear/tangential stress, which is exposed to the cell membrane while they are traversing the micropores at high flow rates^30^. This may result in a higher amount of pore formation and plasma membrane damage, leading to homeostatic imbalance and further cell death pathway activation^31^.

Next, we tested the influence of the various buffers on delivery outcomes resulting from the MFP platform using PC membranes. As mentioned in previous reports, Na^+^-rich physiological buffers (e.g., DPBS) and cell media (physiological buffer plus nutrients, such as DMEM and optiMEM) are the most popular buffer options for intracellular delivery^32^. Therefore, HeLa cells were processed under the optimized flow rate, while each time, they were resuspended in one of these delivery buffers. The results indicated better delivery outcomes when the cells were resuspended in DPBS as it has been revealed a high level of Ca^2+^ and Na^+^ ions in this buffer can significantly increase the rate of resealing plasma membrane disruptions (Supplementary Fig. 2)^33^.

### Intracellular delivery via conventional electroporation and lipofection

To compare the delivery performance of the MFP approach with benchtop options, we electroporated HeLa cells under similar concentrations after optimizing the Neon system with its special delivery buffer. We achieved about 85% loading efficiency of 70 kDa FITC-dextran, while ~ 78% of HeLa cells were viable after inducing one pulse of 1.5 kV for the duration of 30 ms according to the manufacturer’s protocol (Supplementary Fig. 3A, B). Although better delivery efficiencies can be achieved using higher voltages, a rapid drop in the cell viability would occur when voltage pulses go beyond 1.5 kV with longer durations. After optimizing the delivery conditions using Lipofectamine™ 3000, we obtained ~ 25% efficiency in loading H2B-GFP plasmid DNA inside the HeLa cells (Supplementary Fig. 3C, D).

### MFP using SiN microsieves

**Figure 2:**
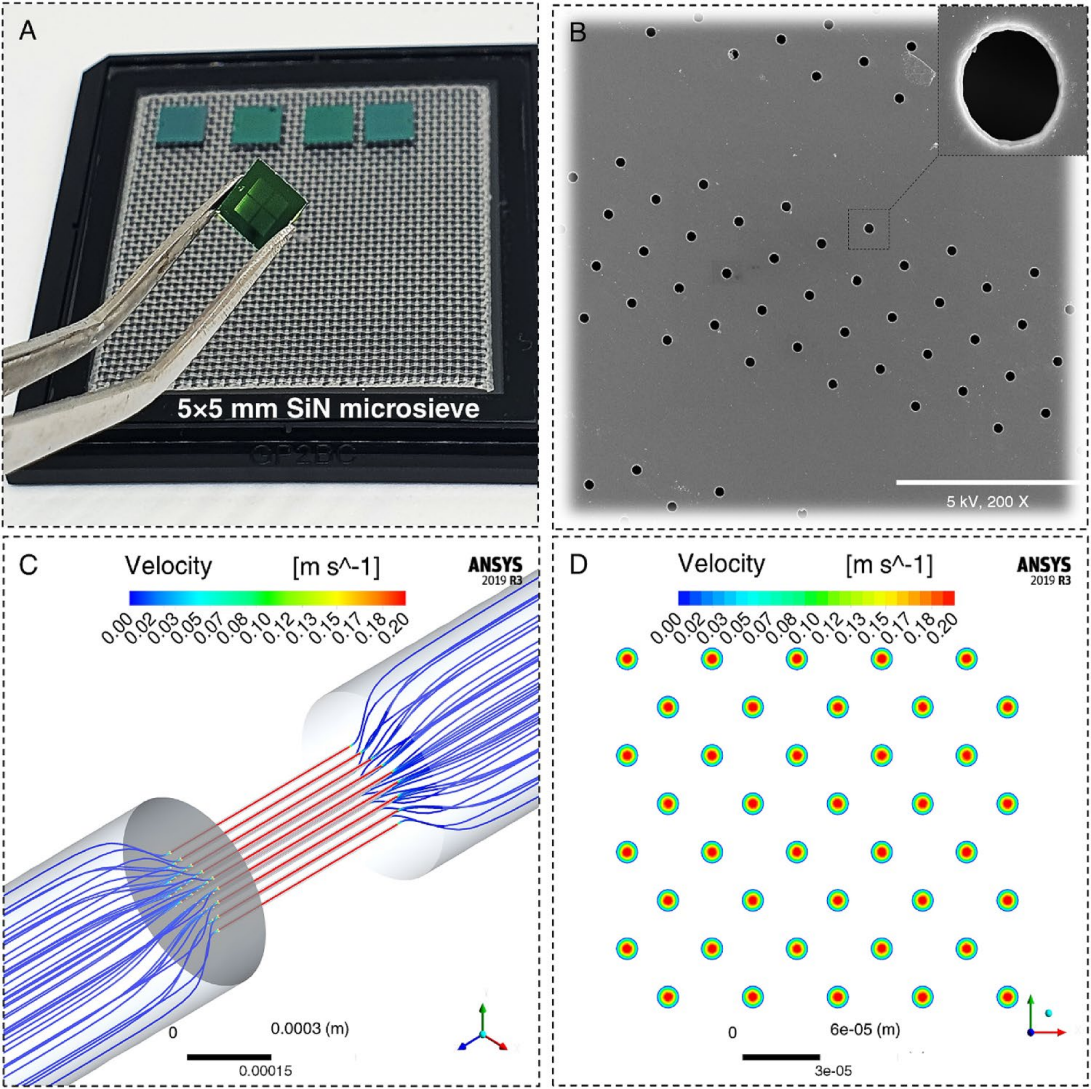
MFP microsieves and numercial analysis. (**A**) Unmounted SiN microsieves. (**B**) Each microsieve membrane is 5 × 5 mm containing 14 fields with 37% porosity increasing the throughput of the MFP approach (10^7^ cell min^-1^). Scale bar indicates 100 µm. (**C**, **D**) These plans show the 3D illustration of streamlines passing through the microsieves with the inlet velocity of 0.24 m s^−1^ and the velocity contour of the cross-sectional area for 40 micropores, respectively. Figure (**C**, **D**) were generated using ANSYS 2019R3 (https://www.ansys.com/academic/students/ansys-student).

**Figure 3:**
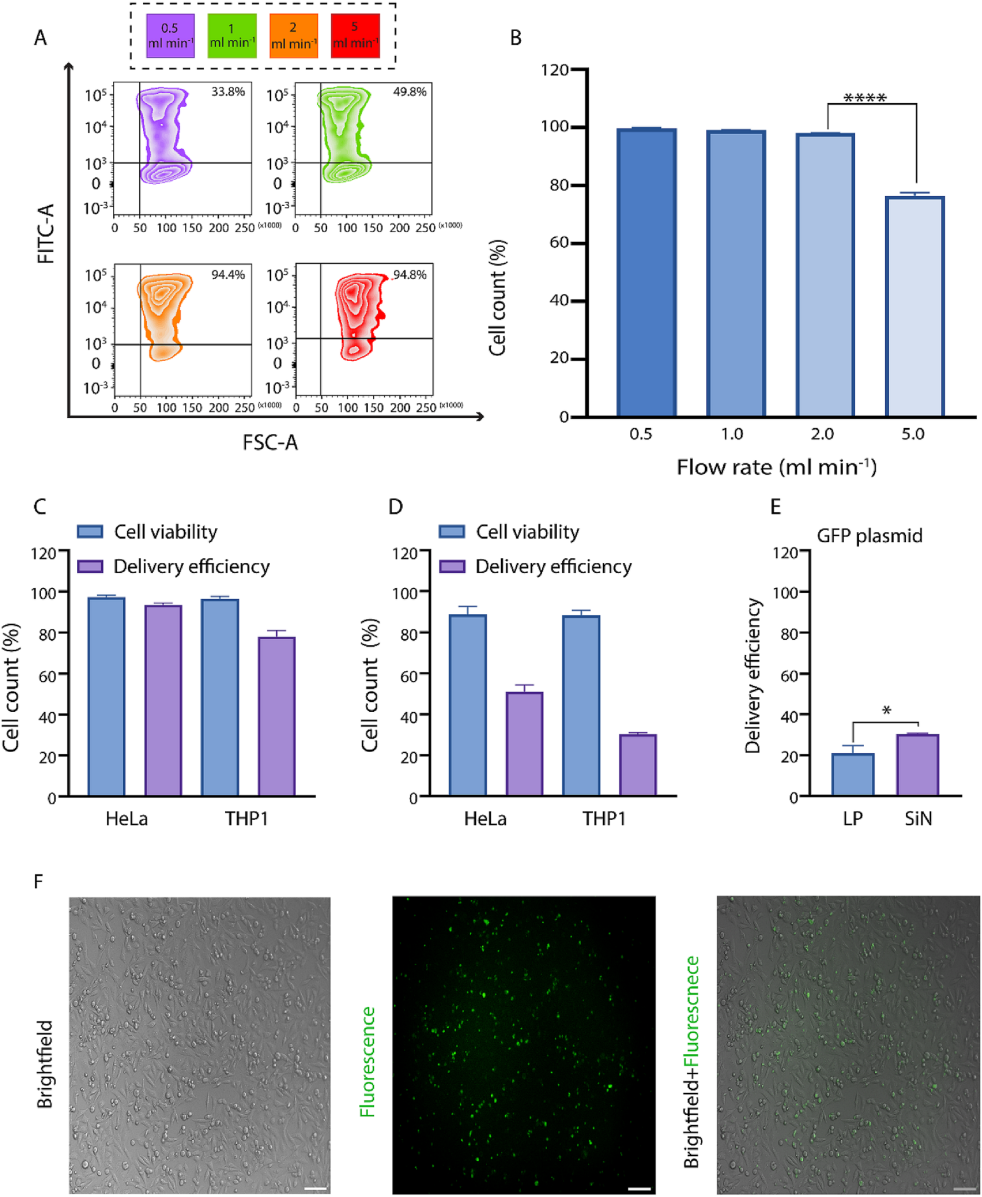
Delivery performance of the MFP setup with SiN microsieves. (**A**) The optimal flow rate was defined at 2 ml min^−1^, in which the cells demonstrated the highest rate of cell viability (~ 98%) and delivery efficiency (94.4%). (**B**) The viability of cells that traversed the SiN micropores showed a sharp decrease at the flow rate of 5 ml min^−1^. ****Indicate the *P*-value < 0.0001. (**C**, **D**) These bar plots display successful delivery of 70 kDa (**C**) and 2000 kDa (**D**) FITC-dextran molecules into both adherent (HeLa) and suspension (THP1) cell lines. (**E**) This bar plot demonstrates the successful loading of plasmid DNA encoding GFP-tagged histone H2B into the HeLa cells. LP: Lipofection, SiN: MFP setup with SiN membranes. *Indicate the *P*-value < 0.05. (**F**) Brightfield, fluorescent, and merged images of HeLa cells 18 h after processing via MFP using SiN microsieves. Scale bar represents 100 µm. All error bars represent the mean ± standard error of the mean, N = 3.

Previous studies have shown the application of the SiN microsieves in the label-free isolation of circulating tumor cells from the whole blood^34^ as well as the detection and filtration of bacteria in biological fluids^35,36^. In this study, for the first time, we hypothesized that these ultra-thin SiN microsieves (Fig. 2A, B) with high mechanical strength (up to 0.5 MPa), biocompatibility, monodisperse pores, and uniform pore size and shape could be used to induce membrane disruptions efficiently^26,37,38^. MFP treatment using these microsieves will result in membrane permeabilization and improve the delivery outcomes compared to the MFP setup with commercially available track-etched PC membranes. Prior to undertaking any experiments on the delivery performance of the MFP using these microsieves, a numerical simulation of fluid flow passing through the SiN membranes was performed to predict the fluid flow behavior and ensure the feasibility of the experiment considering the tiny size of the microsieves and the pressure generated by passing through the micropores. The simulation results that are shown in Fig. 2C, D indicate a laminar flow with the inlet velocity of 0.24 m s^−1^, which corresponds to the flow rate of 2 ml min^−1^ in our experimental setup. To reduce the computational load of the simulation, 40 micropores were modeled, and a mesh and domain size independence check was conducted to ensure that this simplification is not affecting the simulation results. The sample flow velocity was a crucial parameter in our experiments as the low velocity resulted in cell sedimentation above the filter, followed by filter blockage. While a higher velocity overcame the problem of clogging and increased the throughput, it induced critical damages to the cells due to the excessive stress and vortices generated in the flow. To compare the delivery performance of the MFP setup with commercially available track-etched filters (porosity 5–20%) and SiN microsieves (porosity 37%), the optimal delivery buffer (DPBS) was used in both conditions. As such, we just tried to optimize the fluid flow velocity by passing a mixture of HeLa cells and 70 kDa FITC-dextran through the MFP setup under a range of flow rates between 0.5 to10 ml min^−1^. As the cells were traversing the micropores, they were stretched and partially deformed, leading to rupture formation in the plasma membrane and diffusive uptake of FITC-dextran molecules inside the cells. In line with our expectations, the flow cytometry results indicated a monotonic increase in delivery efficiency by increasing the operational flow rate (Fig. 3A). However, the cell viability revealed a sharp decline at the flow rates higher than the 2 ml min^−1^ (Fig. 3B). Thus, we defined the optimal delivery condition of the MFP platform when the cells were resuspended in DPBS as a delivery buffer, and they were passing through the SiN microsieves under the flow rate of 2 ml min^−1^. In contrast to the MFP with PC membranes, forcing HeLa cells through these microfabricated filters at this optimal flow rate, we achieved a significantly higher level of 70 kDa FITC dextran internalization (94.4% delivery efficiency with 98% cell viability), which can be attributed to the higher rigidity and porosity of these microsieves (Fig. 3C).

### MFP using SiN microsieves can load different cargoes into different cell types

Next, to expand the applicability of this delivery method, we forced THP1 suspension cells through the SiN microsieves to load 70 kDa FITC-dextran under fixed flow conditions. We used THP1 monocytic model cells to provide further insight into the potential of the MFP setup using these microsieves for cancer immune therapy purposes. It is interesting to note that treated THP1 cells exhibited comparable cell viability (97.7%) but different delivery efficiency (~ 80%) (Fig. 3C) that can stem from the intrinsic mechanical properties (e.g., stiffness) of different cell types^39^.

To expand the applicability of this method in loading nanomaterials with different sizes, we delivered 2000 kDa FITC-dextran (large-sized FITC-dextran, ~ 55 nm) and GFP-tagged H2B encoding plasmid DNA into two different cell lines mentioned above. We designed an MFP experiment using the SiN microsieves to load 2000 kDa FITC-dextran into the HeLa cells under the optimal delivery conditions. As presented in Fig. 3D, the results indicated a delivery efficiency of 54.7% in loading 2000 kDa FITC-dextran inside the HeLa cells while they demonstrated up to 91.5% cell viability. Moreover, using this delivery platform, up to 31.1% of THP1 cells (90.8% cell viability) were loaded with 2000 kDa FITC-dextran. These results elaborated on the applicability of this platform for the efficient delivery of extremely large biomolecules into the cells of interest. In addition to the cytoplasmic delivery of small nanomaterials, we also investigated the feasibility of this platform in gene delivery as it is still challenging to navigate the large-sized plasmid DNA (~ 100–200 nm) through the plasma membrane. For this purpose, we tried to load 5.1 kb plasmid DNA encoding fluorescent histone H2B protein into HeLa cells. As shown in Fig. 3E, we could successfully achieve 30.8% loading efficiency of plasmid DNA and 90.4% cell viability according to the flow cytometry results. These results were confirmed by fluorescent microscopy (Fig. 3F) as a higher number of HeLa cells expressed GFP-tagged histone H2B protein 24 h post-delivery compared with those transfected using the conventional lipofection.

**Figure 4:**
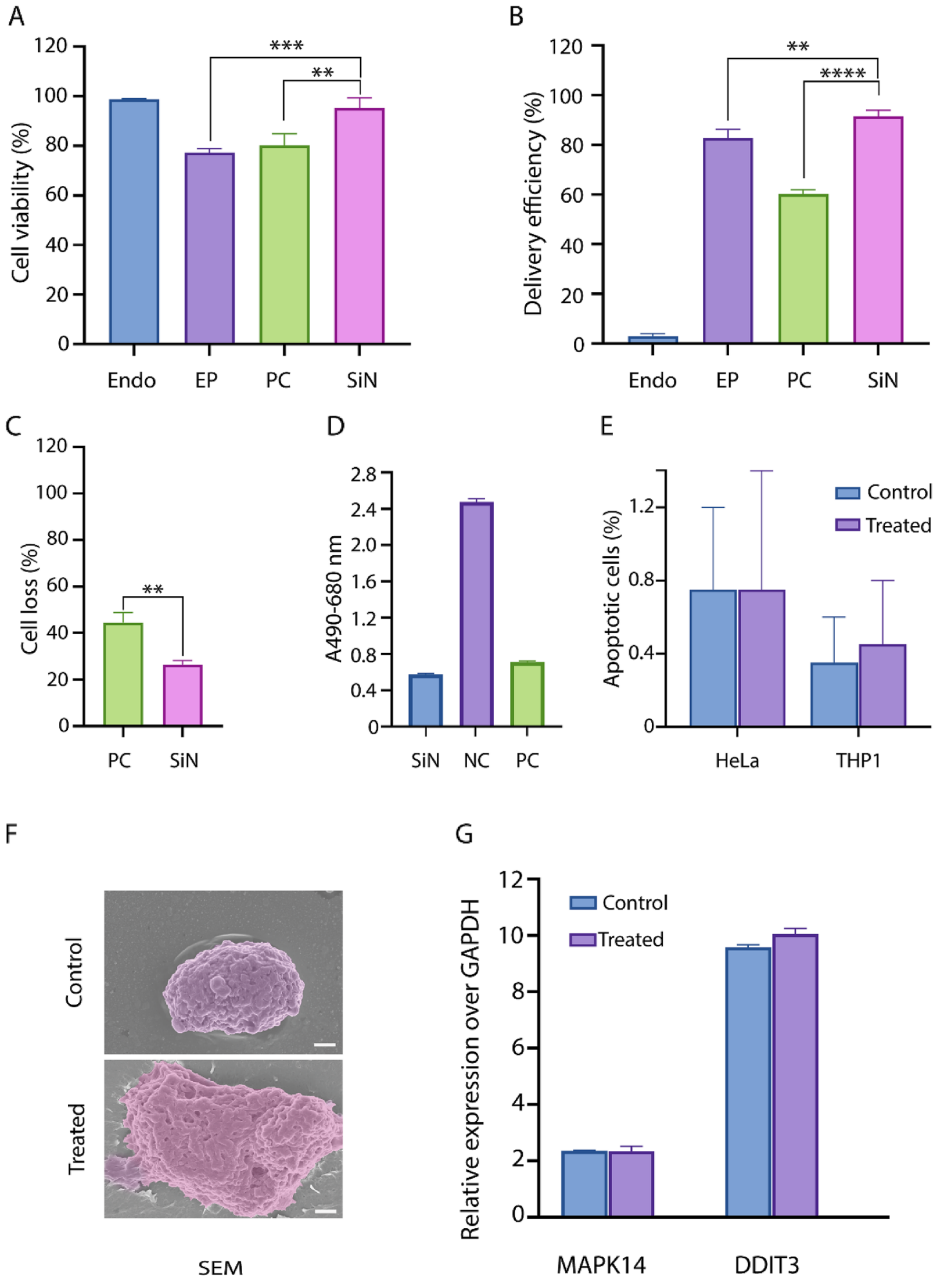
Comparing the delivery performance of the MFP setup using SiN microsieves with the mainstream options. Treated HeLa cells using SiN filters indicated higher cell viability compared with those treated by PC filters and electroporated cells (**A**). ** and ***Indicate the *P*-value < 0.01 and *P*-value < 0.001, respectively. The delivery efficiency of loading 70 kDa FITC-dextran was significantly higher when SiN microsieves were used in the MFP system (**B**). ** and ****Indicate the *P*-value < 0.01 and *P*-value < 0.0001, respectively. (**C**) In the MFP setup, cell loss is significantly higher when the PC membranes were used. **Indicate the *P*-value < 0.01. (**D**) LDH assay demonstrated the minimal membrane leakage from the cells forced through the SiN microsieves. (**E**) Annexin V binding assay of control and MFP treated cells indicated negligible cell death 18–24 h post treatment. (**F**) SEM analysis of the cells before and after the MFP with SiN filters demonstrated morphological change and pore formation in the plasma membrane. Scale bar represents 2 µm. (**G**) qPCR analysis revealed no significant difference between the expression of stress-related genes, MAPK14 and DDIT3, between untreated and microfiltroporated cells using the SiN microsieves.

Our findings confirmed that the MFP setup with SiN microsieves resulted in improved delivery efficiency with higher cell viability and lower cell loss under similar conditions (Fig. 4A–C). Since this difference has not been found elsewhere, it is probably due to the high rigidity and porosity of these microsieves with monodispersed pores that induce almost equal shear stress to the individual cells. Moreover, as these microsieves are thinner than the commercially available track-etched filters, the cells may require less time to traverse the micropores, which might be considered an important factor to achieve better delivery and cell health outcomes.

### MFP induce minimal damage to the plasma membrane

Finally, as the last step of our investigation, it was essential to analyze the cell membrane injury and DNA damage induced by the MFP setup using SiN microsieves. Accordingly, the LDH activity assay, SEM imaging, confocal microscopy, and qPCR of DNA damage indicator genes were performed to evaluate the effects of this treatment on the plasma membrane and nuclear integrity. LDH is one of the key cytoplasmic enzymes actively involved in the reversible conversion of lactate into pyruvate^40^. Upon plasma membrane disruption, the LDH enzyme is released from damaged cells into the extracellular environment. Previous studies have reported that the presence of the LDH enzyme in the extracellular environment can serve as a marker of plasma membrane injury and cell death^41,42^. The membrane leakage assay of control and microfiltroporated cells via these ultra-thin membranes indicated no significant difference in the LDH level 24 h post-delivery (Fig. 4D). Moreover, Annexin V (Annexin A5) binding assay of control and MFP treated cells demonstrated negligible cell death via apoptosis for two tested cell lines (Fig. 4E). Therefore, we noted that this setup might be considered as one of the safe delivery options that is gentle on the plasma membrane and significantly decrease cellular damage. Accordingly, confocal microscopy of HeLa cells post-delivery demonstrated cytoskeletal and nuclear envelope integrity (Supplementary Fig. 3E). Similarly, the pore creation in plasma membrane induced by MFP platform using microsieves was also analyzed through the SEM analysis. The HeLa cells before and after passing through the microsieves under the optimal delivery conditions were fixed on a coverslip and coated for SEM imaging, which demonstrated the formation of multiple pores in the plasma membrane for efficient cargo passage while its integrity was preserved (Fig. 4F). Furthermore, bright field microscopy of HeLa cells 1 and 5 days post-delivery confirmed that this procedure does not adversely affect the cell growth (Supplementary Fig. 3F, G). In addition, qPCR for the DNA damage indicator genes, DDIT3 and MAPK14, was carried out. DDIT3 gene, also called growth arrest and DNA damage-inducible 153 (GADD153), plays a key role in regulating the response to a wide variety of cellular stresses (i.e., endoplasmic reticulum stress) and DNA damage^43,44^. The transcriptional activity of this gene is regulated by the MAPK14 gene (also known as p38-α), which is activated in response to the cellular stress conditions^45^. No significant difference was observed in the expression of DDIT3 and MAPK14 genes between the control and microfiltroporated cells (Fig. 4G). Hence, it can be concluded that MFP using SiN microporous membranes, cells are capable to uptake the exogenous nanomaterials of different sizes while minimal damage was induced to the plasma membrane.

## Conclusion

In this study, we presented a delivery platform that can efficiently load nanomaterials into the cells of interest. In this work, for the first time, we used microengineered SiN filters with round-shape uniform micropores in the MFP setup to load various nanomaterials, including FITC-dextran and plasmid DNA, into different cell types. Due to the outstanding mechanical property of these microporous membranes, the cells experience minimal membrane damage compared with those treated with commercially available track-etched PC filters. The uniformity of micropores in SiN membrane filters can increase the number of membrane discontinuities in target cells leading to an increased level of cargo efflux while maintaining a high level of cell viability. In contrast to PC filters, MFP setup with microengineered SiN filters is characterized by higher delivery efficiency and minimal cell damage while achieving a low-cost, rapid, and user-friendly operation. Nanomaterial delivery using SiN membranes does not require any external power supply, and the only required technology is a syringe pump. A further investigation can evaluate the effect of membrane rigidity, porosity, pore size, and shape on delivery outcomes. In conclusion, we envision that the MFP approach using SiN microporous membranes with promising delivery performance compared to the alternative methods would represent a potential biocompatible and highly efficient delivery approach for loading nanomaterials inside a wide range of cell types.

## Supplementary Information

Supplementary Information.
