# Supplementary material for: Microengineered filters for efficient delivery of nanomaterials into mammalian cells
Source: Sci Rep. 2022 Mar 14;12:4383. doi: 10.1038/s41598-022-08300-2 (PMC8921284; doi:10.1038/s41598-022-08300-2)
Supplement: Supplementary file 1 — Supplementary Information. [file 41598_2022_8300_MOESM1_ESM.docx]

**Microengineered Filters for Efficient Delivery of Nanomaterials into Mammalian Cells**

Dorsa Morshedi Rad1, Meysam Rezaei2,3,4, Payar Radfar1, Majid Ebrahimi Warkiani1,3,4,5*

1School of Biomedical Engineering, University of Technology Sydney, Sydney, New South Wales 2007, Australia

2Genea, Sydney, New South Wales 2000, Australia

3Institute for Biomedical Materials & Devices (IBMD), Faculty of Science, University of Technology Sydney, Sydney, NSW 2007, Australia

4SUStech-UTS Joint Research Centre for Biomedical Materials and Devices, Southern University of Science and Technology, Shenzhen 518055, People’s Republic of China

5Institute of Molecular Medicine, Sechenov University, Moscow, 119991, Russia

* Contact:

Majid Ebrahimi Warkiani ([majid.warkiani@uts.edu.au](mailto:majid.warkiani@uts.edu.au))

School of Biomedical Engineering, University Technology Sydney, New South Wales 2007, Australia


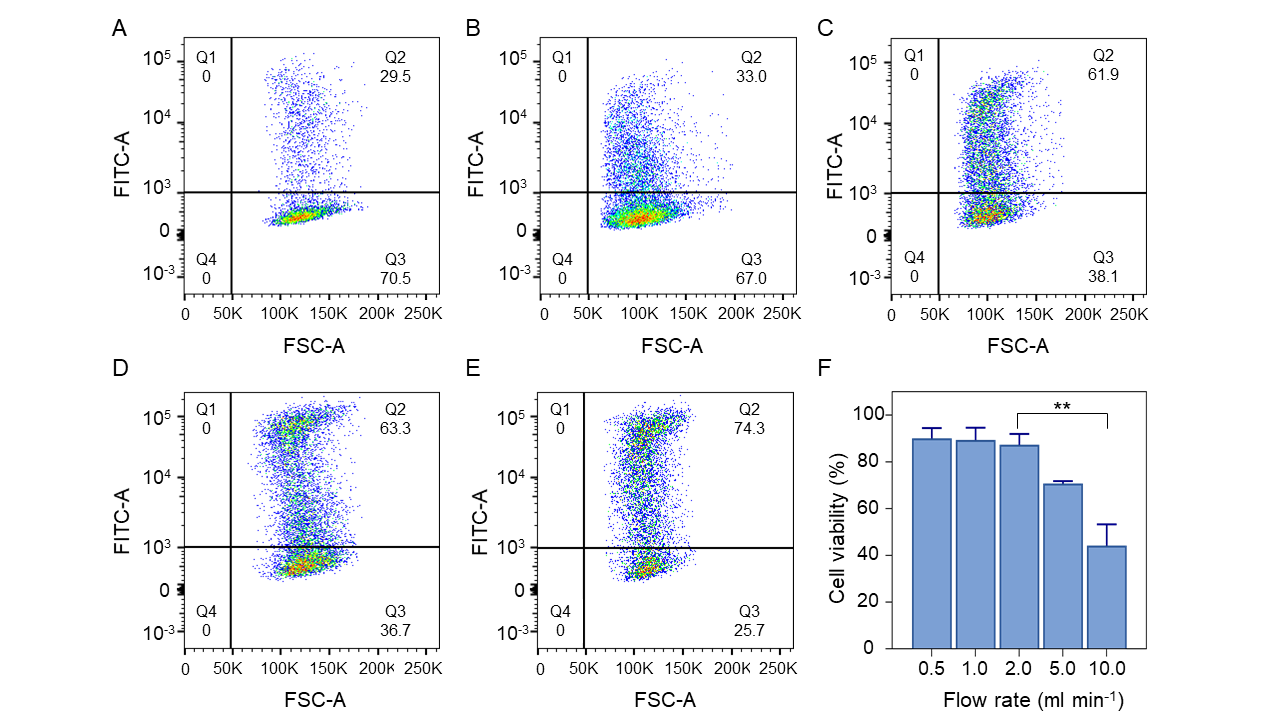
**Supplementary Information**

# **Supplementary Figure 1:** Testing the operational flow rate from 0.5-10 ml min^-1^. A-E) these plans indicate the delivery efficiency at the flow rate of 0.5, 1, 2, 5, and 10 ml min^-1^, respectively. The best delivery results were achieved at the flow rate of 2 ml min^-1^ with 61.9% delivery efficiency and 84% cell viability. F) Cell viability of treated cells under different tested flow rates. All error bars represent the mean ± standard error of the mean (N = 3) and ** indicate P-values below 0.01.

#
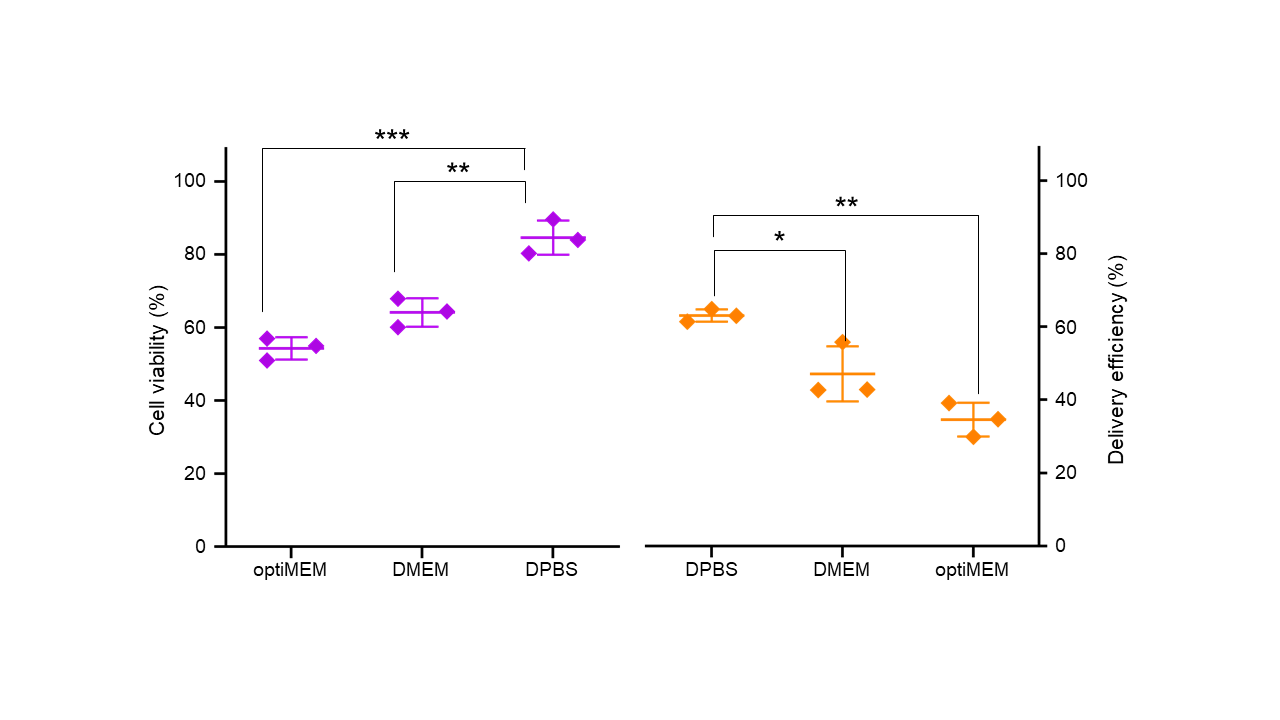
**Supplementary Figure 2:** The Scatter plots represent that at the optimal flow rate, the highest cell viability and delivery efficiency were achieved when DPBS was used as a delivery buffer. All error bars indicated the mean ± standard deviations (N = 3) and *, **, and *** indicate P-values below 0.01, 0.05, and 0.001, respectively.

# **
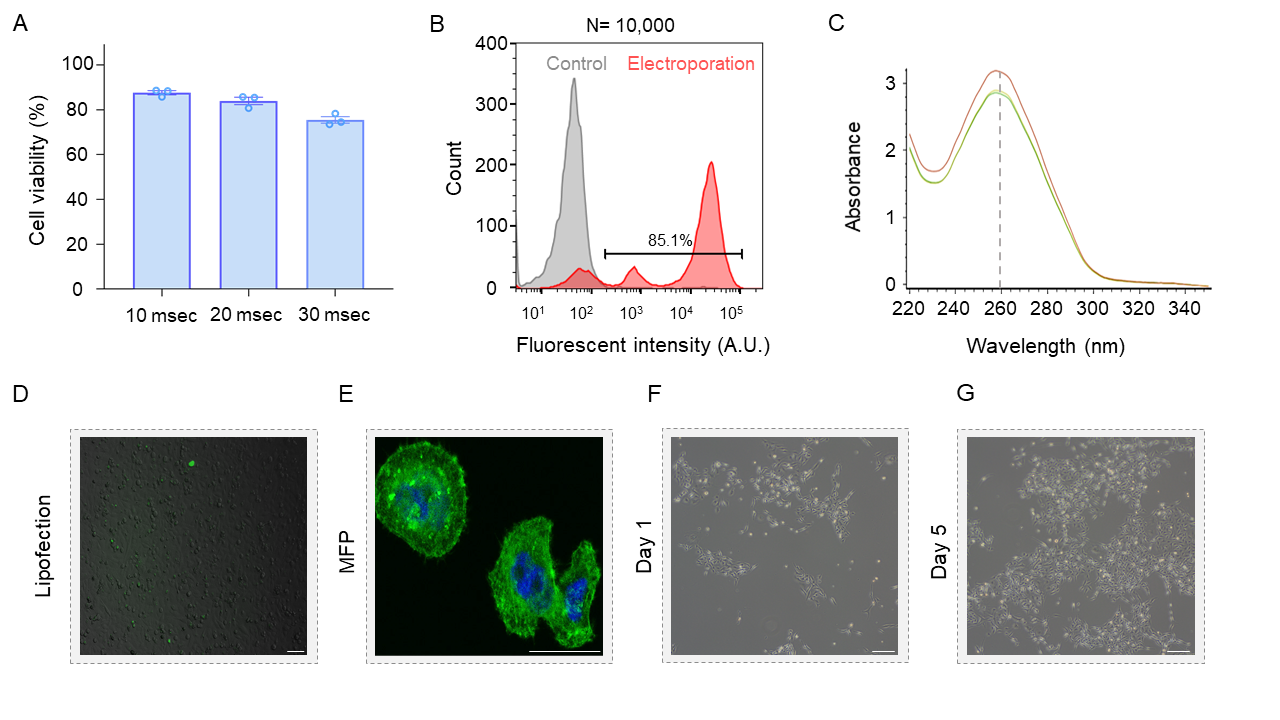
 Supplementary Figure 3:** A) Viability of HeLa cells experienced electric pulses with the strength of 1.5 kV for the durations of 10, 20, and 30 msec. B) Among the tested conditions, an electric shock with the voltage of 1.5 kV for the duration of 30 msec resulted in about 85% efficiency of loading 70 kDa FITC dextran into HeLa cells while they were ~78% viable. C) Absorbance measurement indicated the high yield and purity of the GFP plasmid DNA. D) HeLa cells loaded with H2B GFP plasmid DNA via lipofection. Each green dot represents a cell expressing GFP-tagged H2B. The scale bar represents 100 µm. E) Confocal microscopy of HeLa cells passed through the SiN membranes confirmed cytoskeletal and nuclear envelope integrity of processed cells. The scale bar represents 20 µm. Bright field microscopy of processed HeLa cells via SiN membranes after 1 (F) and 5 days (G) demonstrated that cell growth is not affected by the delivery procedure. The scale bar represents 200 µm.
